# Ocular findings in patients with acquired ATTRv amyloidosis following domino liver transplantation

**DOI:** 10.1371/journal.pone.0291716

**Published:** 2023-09-15

**Authors:** Junya Kitahara, Tsuneaki Yoshinaga, Shinji Kakihara, Takao Hirano, Akira Imai, Teruyoshi Miyahara, Masahide Yazaki, Yoshiki Sekijima, Toshinori Murata

**Affiliations:** 1 Department of Ophthalmology, Shinshu University School of Medicine, Nagano, Japan; 2 Department of Medicine (Neurology & Rheumatology), Shinshu University School of Medicine, Nagano, Japan; 3 Institute for Biomedical Sciences, Shinshu University, Nagano, Japan; 4 Clinical Laboratory Sciences Division, Shinshu University Graduate School of Medicine, Nagano, Japan; Yokohama City University, JAPAN

## Abstract

**Purpose:**

To investigate the presence of amyloidosis-related ocular findings in patients who received domino liver transplantation from ATTRv amyloidosis donors.

**Methods:**

We reviewed the ocular findings in patients who had previously undergone domino liver transplantation and received ophthalmologic examinations between January 2009 and March 2023. The presence of amyloidosis-related ocular findings was retrospectively assessed by two ophthalmologists.

**Results:**

During the study period, a total of 7 patients with 14 eyes were examined. All patients were considered as acquired ATTRv amyloidosis. The mean age at the final visit was 64.6±8.4 years (52–75 years), and the mean time since domino liver transplantation was 167.6±76.2 months (69–257 months). The two evaluators’ assessments for amyloidosis-related ocular findings were completely identical. No amyloid fibril deposition was observed in the pupil, lens, or vitreous. Five patients (10 eyes) had a Schirmer test result of 5mm or less than 5 mm, and four patients with a total of 8 eyes underwent fluorescein angiography and indocyanine green angiography, and no evidence of retinal amyloid angiopathy was found on fluorescein angiography. However, three patients with 6 eyes showed choroidal amyloid angiopathy on indocyanine green angiography.

**Conclusion:**

While cases of choroidal amyloid angiopathy were observed, serious amyloidosis-related ocular complications such as vitreous opacity or secondary glaucoma did not occur even in the long term after domino liver transplantation.

## Introduction

Hereditary transthyretin amyloidosis is a disease caused by genetic abnormalities in the gene that codes for transthyretin (TTR), resulting in the production of variant TTR and systemic amyloidosis [1]. Previously known as familial amyloid polyneuropathy, this disease is now recommended to be called ATTRv amyloidosis by the International Society of Amyloidosis [2]. TTR is mainly produced in the liver, where it forms a tetramer in the plasma and functions as a transporter for retinol, but also for thyroxine [3]. The presence of variant TTR destabilizes the tetramer, leading to protein misfolding and an increased propensity for amyloid fibrils formation [3]. Typically, this disease begins with neuropathy in adulthood and has a fatal outcome within about 10 years without treatment, but effective therapies such as liver transplantation and siRNA therapy have extended life expectancy and improved quality of life [4–7]. As a result, ocular complications such as vitreous amyloidosis and secondary amyloid glaucoma have become significant clinical problems, as TTR is also produced in the eye, especially in the pigment epithelium of the eye [8–12].

Liver transplantation has been performed for ATTRv amyloidosis since 1990 [13] and has now been established as an effective treatment [14]. However, due to a shortage of liver transplant donors for patients with liver failure, domino liver transplantation, in which the liver of a patient with ATTRv amyloidosis is transplanted into a patient with liver failure, has become common [15]. Although many cases have been reported in which the diagnosis of amyloidosis was made after domino liver transplantation [16,17], whether these patients develop ocular complications due to acquired ATTRv amyloidosis has not been sufficiently investigated. According to the sole previous paper regarding ocular complications in this setting, no apparent amyloidosis-related ocular complications were observed six years after domino liver transplantation [18]. However, ocular complications of ATTRv amyloidosis often appear after systemic amyloidosis, and Ando et al. reported that pupillary shape abnormalities, vitreous amyloidosis, and secondary glaucoma typically appear after an average of 5.6 years, 9.5 years, and 14.5 years, respectively, from the onset of systemic amyloidosis [19, 20]. Therefore, it is not clear from the previous report whether patients who received a domino liver transplant really do not develop ocular amyloidosis. Therefore, in the current study, we retrospectively investigated cases with longer follow-up periods to examine the development of ocular amyloidosis.

## Results

During the study period, seven patients with a total of 14 eyes received ophthalmologic examinations at our hospital. All cases have been followed up as acquired ATTRv amyloidosis after confirmation of amyloid fibril deposition by tissue biopsy with Congo red staining. The average age at the last visit was 64.6±8.4 (range: 52–75) years, and the mean time elapsed since domino liver transplantation was 167.6±76.2 (range: 69–257) months. The underlying diseases of the patients were as follows: one case of adult-type citrullinemia, two cases of cirrhosis and hepatocellular carcinoma, one case of cirrhosis, one case of cirrhosis with hepatic encephalopathy, one case of primary sclerosing cholangitis, and one case of primary biliary cirrhosis.

**Fig 1:**
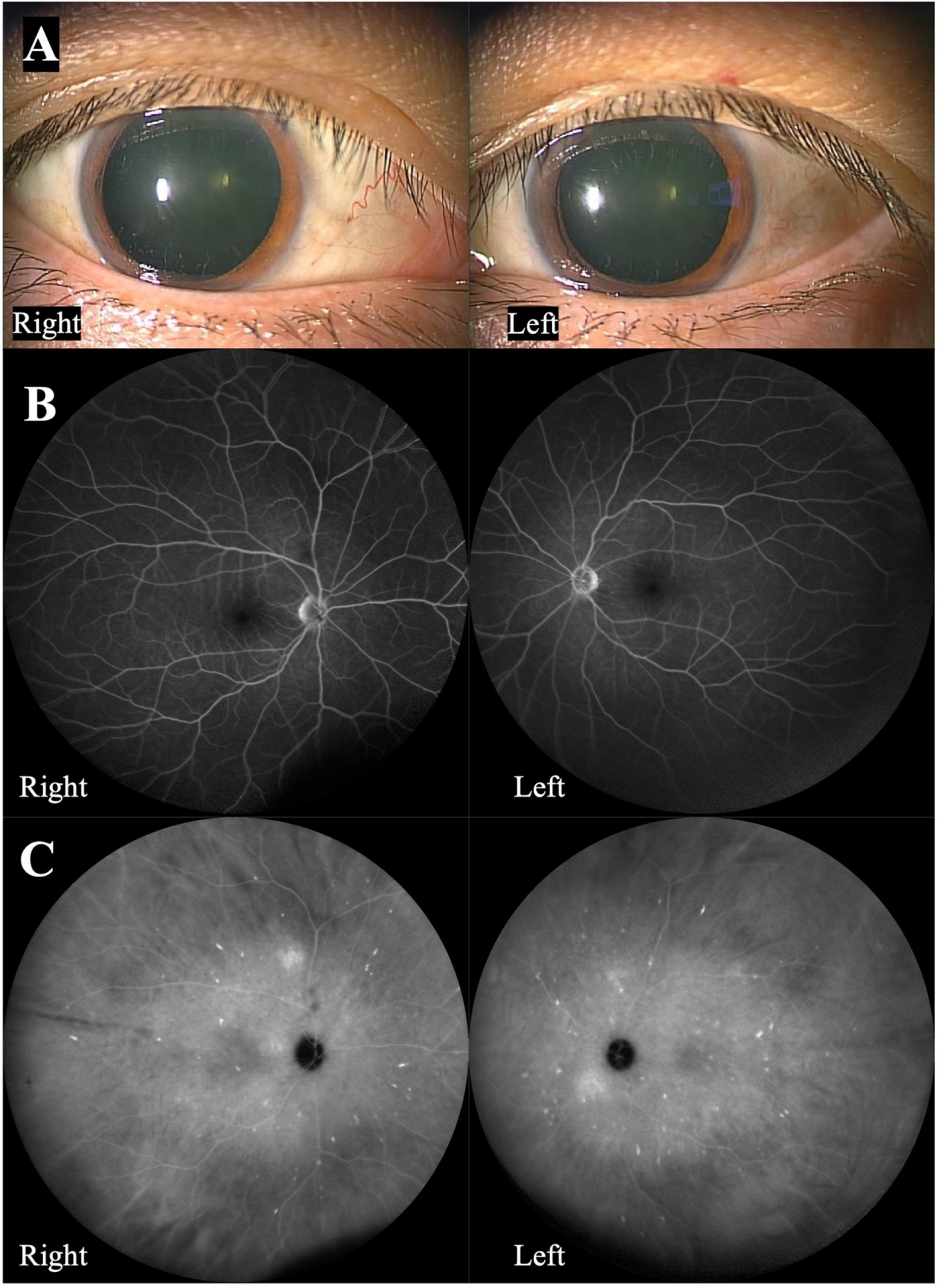
A representative case exhibiting typical findings of choroidal amyloid angiopathy. (A) Slit-lamp microscopy shows that there are no abnormalities in the pupillary shape and amyloid deposition in the lens. (B) Fluorescein angiography shows that there is no retinal amyloid angiopathy. (C) Indocyanine green angiography in the later phase shows that there are several hot spots, indicating choroidal amyloid angiopathy (CAA).

The two ophthalmologists’ assessments for amyloidosis-related ocular findings were completely identical. No abnormalities in the pupillary shape, amyloid deposition on the lens, vitreous opacities, or signs of glaucoma secondary to amyloidosis were observed in any of the 14 eyes. Schirmer’s test showed decreased tear production of 5mm or less than 5 mm in 5 patients with a total of 10 eyes. Four patients with a total of 8 eyes underwent fluorescein angiography and indocyanine green angiography, and no evidence of retinal amyloid angiopathy was found on fluorescein angiography in all eyes. However, three patients with a total of 6 eyes showed Grade 1 choroidal amyloid angiopathy (CAA) on indocyanine green angiography in the late phase (**Fig 1**). In addition, two patients with a total of 3 eyes had open-angle glaucoma, which didn’t seem to be related to amyloidosis. One patient with one eye had central serous chorioretinopathy, which was thought to be associated with systemic steroid use. One patient with a total of 2 eyes had proliferative diabetic retinopathy. **Table 1** shows a summary of the results.

**Table 1:** Demographic and clinical characteristics of seven patients with a total of 14 eyes.

Case. No	Age	Sex	Underlying disease requiring liver transplantation	TTR genotype of liver donor	Follow-up period after domino liver transplantation (months)	Comorbid systemic diseases	Laterality	Decreased tear production	Pupillary shape abnormalities	Amyloid fibril deposition on the lens	Vitreous opacities	Secondary glaucoma	IOP (mmHg)	Retinal amyloid angiopathy	Choroidal amyloid angiopathy	BCVA	Comorbid eye diseases
1	52	M	Adult-type citrullinemia	p. TTRV30L	237	N.A.D.P.	R	+	−	−	−	−	10	−	−	1.2	OAG
							L	+	−	−	−	−	10	−	−	1.2	OAG
2	67	M	Cirrhosis	p. TTRV30M	257	Diabetes	R	+	−	−	−	−	9	−	+	1.2	None
							L	+	−	−	−	−	11	−	+	1.2	None
3	72	F	Cirrhosis and hepatocellular carcinoma	p. TTRV30M	235	Alzheimer’s disease	R	+	−	−	−	−	13	−	+	1.2	OAG
							L	+	−	−	−	−	15	−	+	1.2	None
4	69	M	Cirrhosis with hepatic encephalopathy	p. TTRV30M	69	Diabetes	R	−	−	−	−	−	13	N.A.	N.A.	1	PDR
							L	−	−	−	−	−	15	N.A.	N.A.	0.1	PDR
5	57	F	Primary sclerosing cholangitis	p. TTRV30M	168	N.A.D.P.	R	+	−	−	−	−	11	−	+	1.2	None
							L	+	−	−	−	−	11	−	+	1.2	None
6	75	F	Cirrhosis and hepatocellular carcinoma	p. TTRV30M	111	Graves’ disease	R	+	−	−	−	−	13	N.A.	N.A.	1.5	None
							L	+	−	−	−	−	14	N.A.	N.A.	1.5	None
7	60	F	Primary biliary cirrhosis	p. TTRV30M	96	N.A.D.P.	R	−	−	−	−	−	14	N.A.	N.A.	1.2	None
							L	−	−	−	−	−	16	N.A.	N.A.	1	CSC

TTR, transthyretin; IOP, intraocular pressure; BCVA, best-corrected visual acuity; M, Male; F, Female; N.A.D.P., nothing attributable to the disease process; R, right; L, light; N.A., Not Available; OAG, open angle glaucoma; PDR, proliferative diabetic retinopathy; CSC, central serous chorioretinopathy.

## Discussion

We hypothesized that variant TTR derived from the transplanted liver of ATTRv amyloidosis patients might affect the eye. However, contrary to our expectations, even after up to 21 years post-transplantation, no specific ocular complications of ATTRv amyloidosis, such as pupillary shape abnormalities or vitreous opacities, were observed. However, on indocyanine green angiography, hot spots in the late phase were observed in 3 cases (6 eyes), indicating CAA. Decreased tear production is also one of the possible manifestations of ATTRv amyloidosis, which can occur due to autonomic nerve dysfunction or amyloid deposition in the lacrimal glands [21,22]. In this study, decreased tear production was confirmed by a Schirmer test in 10 eyes of 5 patients. However, due to the lack of pathological examination and the low specificity of decreased tear production for ATTRv amyloidosis, it is uncertain whether this is caused by acquired ATTRv amyloidosis or not. Overall, these results suggest that variant TTR, which is derived from the transplanted liver and exists in the plasma, may have little impact on ocular findings but deposit in the choroid.

The previous literature has reported that analyses of ocular amyloidosis in patients with ATTRv amyloidosis after long-term liver transplantation revealed that only the choroidal tissue was affected in the eye by the transplanted liver with a relatively high proportion of wild-type TTR compared to other intraocular tissues. Because the choroidal capillaries are fenestrated vessels that act as the major blood suppliers for the neuroepithelial layer of the retina, however, retina vessel has a tight junction, which has low permeability with substance [23,24]. Based on this previous report, we hypothesize that in the current cases after domino liver transplantation, the variant TTR from the transplanted liver is impacting the generation of CAA, which we believe highly compatible considering the previous literature [25]. The previous case report, which reports mutant transthyretin cannot cross the blood-eye barrier, also supports the results of this study [26].

These reports might support the fact that even in the era of effective systemic therapy, patients with ATTRv amyloidosis may develop amyloidosis-related eye complications [8]. That is, effective systemic therapies are treatments targeting the liver, their inhibitory effects on variant TTR production in the pigment epithelium [9,10] is low, and amyloid oculopathy characterized by pupillary shape abnormalities, amyloid deposition in the lens, vitreous amyloidosis, and secondary glaucoma characterized by high intraocular pressure [27,28] cannot be suppressed in patients with ATTRv amyloidosis.

This study suggested that patients who underwent domino liver transplantation may not need to pay excessive attention to amyloid oculopathy, but data supports that amyloid fibrils that cause amyloid oculopathy in ATTRv amyloidosis cannot be resolved by systemic therapy alone and require eye-specific treatment.

Several groups have reported that the severity of CAA may be a biomarker for systemic amyloidosis severity [29,30], and this study supports the idea that CAA detected by indocyanine green angiography[31] may reflect amyloid fibrils in the bloodstream.

The present study has several limitations. It is a retrospective case series study, and the number of cases is small. There is no control group. We should also note the fact that not all ATTRv amyloidosis patients develop intraocular amyloid deposits and that one donor was p. TTRV30L and the other six donors were p. TTRV30M in this study. However, since no previous report has evaluated patients who underwent domino liver transplantation in detail after a long-term follow-up period, we believe it is a meaningful study.

In summary, patients after domino liver transplantation did not show specific findings of ATTRv amyloidosis, except for CAA, even after a long-term follow-up period. To the best of our knowledge, this is the first study to describe ocular findings more than 10 years after domino liver transplantation.

## Methods

We retrospectively analyzed patients who received domino liver transplants from ATTRv amyloidosis donors and underwent ophthalmic examinations at the Department of Ophthalmology, Shinshu University School of Medicine between January 2009 and March 2023. Based on medical records, we investigated the underlying diseases that led to liver transplantation and concomitant eye diseases between April 1st, 2023, and May 10th, 2023. Comprehensive ophthalmic examinations, including measurement of best-corrected visual acuity using the Landolt C chart, slit-lamp microscopy, tonometry, and fundus examination with dilation of the pupil, were performed in detail. Authors had access to information that could identify individual participants during or after data collection.

The diagnosis of acquired ATTRv amyloidosis was conducted based on several examinations including tissue biopsy with Congo red staining and the manifestation of symptoms, by amyloidosis specialists from the Department of Medicine (Neurology & Rheumatology), Shinshu University School of Medicine.

Ocular findings, such as retinal amyloid angiopathy and CAA, as well as the presence of amyloidosis-related ocular complications (pupillary shape abnormalities, amyloid fibril deposition on the lens, vitreous opacities, and secondary glaucoma), were retrospectively evaluated independently by two ophthalmologists (J.K. and S.K.) based on several imaging modalities including fundus photographs, optical coherence tomography, fundus fluorescein angiography, indocyanine green angiography, and anterior segment photographs by slit-lamp microscopy. Choroidal amyloid angiopathy (CAA) was judged according to the CAA severity based on the staging system previously described [32]. CAA of each patient was classified as Grade 0, no apparent CAA; Grade 1, punctiform; Grade 2, linear patches; and Grade 3, marked linear, based on the hyper fluorescence extent at the late phase of indocyanine green angiography [32]. We consider glaucoma or an elevated intraocular pressure of 25 mmHg or greater with either characteristic irregularly shaped pupil or amyloid deposition on the lens surface as amyloid glaucoma.

The study was conducted in accordance with the tenets of the Declaration of Helsinki, and the Institutional Review Board of Shinshu University approved the study (Ethics Review Approval Number: 4922). Informed consent was obtained using the opt-out method. Written informed consent from each patient for publication was not required. Instead, we posted the study protocol at the study institutions to notify participants about the study.
